# General Practitioners’ Perspectives About Remote Dermatology Care During the COVID-19 Pandemic in the Netherlands: Questionnaire-Based Study

**DOI:** 10.2196/46682

**Published:** 2023-06-13

**Authors:** Esmée Tensen, Craig Kuziemsky, Monique W Jaspers, Linda W Peute

**Affiliations:** 1 Department of Medical Informatics Amsterdam UMC location University of Amsterdam Amsterdam Netherlands; 2 Amsterdam Public Health Digital Health Amsterdam Netherlands; 3 Ksyos Health Management Research Amsterdam Netherlands; 4 MacEwan University Edmonton, AB Canada

**Keywords:** teledermatology, teledermoscopy, dermatology, dermoscopy, telemedicine, telehealth, remote care, general practitioner, GP, general practice, family physician, COVID-19, questionnaire, perspective, mobile phone

## Abstract

**Background:**

The COVID-19 pandemic affected the delivery of primary care and stimulated the use of digital health solutions such as remote digital dermatology care. In the Netherlands, remote store-and-forward dermatology care was already integrated into Dutch general practice before the COVID-19 pandemic. However, it is unclear how general practitioners (GPs) experienced this existing digital dermatology care during the pandemic period.

**Objective:**

We investigated GPs’ perspectives about facilitators and barriers related to store-and-forward digital dermatology care during the COVID-19 pandemic in the Netherlands, using a sociotechnical approach.

**Methods:**

In December 2021, a web-based questionnaire was distributed via email to approximately 3257 GPs who could perform a digital dermatology consultation and who had started a digital consultation (not necessarily dermatology) in the previous 2 years. The questionnaire consisted of general background questions, questions from a previously validated telemedicine service user satisfaction questionnaire, and newly added questions related to the pandemic and use of the digital dermatology service in general practice. The open-ended and free-text responses were analyzed for facilitators and barriers using content analysis, guided by an 8-dimensional sociotechnical model.

**Results:**

In total, 71 GPs completed the entire questionnaire, and 66 (93%) questionnaires were included in the data analysis. During the questionnaire distribution period, another national lockdown, social distancing, and stay-at-home mandates were announced; thus, GPs may have had increased workload and limited time to complete the questionnaire. Of the 66 responding GPs, 36 (55%) were female, 25 (38%) were aged 35-44 years, 33 (50%) were weekly platform users, 34 (52%) were working with the telemedicine organization for >5 years, 42 (64%) reported that they used the store-and-forward platform as often during as before the pandemic, 61 (92%) would use the platform again, 53 (80%) would recommend the platform to a colleague, and 10 (15%) used digital dermatology home consultation. Although GPs were generally satisfied with the digital dermatology service, platform, and telemedicine organization, they also experienced crucial barriers to the use of the service during the pandemic. These barriers were GPs’ and patients’ limited digital photography skills, costs and the lack of appropriate equipment, human-computer interface and interoperability issues on the telemedicine platform, and different use procedures of the digital dermatology service.

**Conclusions:**

Although remote dermatology care was already integrated into Dutch GP practice before the pandemic, which may have facilitated the positive responses of GPs about the use of the service, barriers impeded the full potential of its use during the pandemic. Training is needed to improve the use of equipment and quality of (dermoscopy) images taken by GPs and to inform GPs in which circumstances they can or cannot use digital dermatology. Furthermore, the dermatology platform should be improved to also guide patients in taking photographs with sufficient quality.

## Introduction

### Background

The COVID-19 pandemic had a major impact on the access and delivery of primary care owing to social distancing and other public health measures, such as lockdowns or stay-at-home mandates [1]. This unprecedented crisis forced health care organizations to consider innovative ways to plan and deliver their care remotely [2] and led to substantial changes in health care delivery. One of those changes has been the rapid growth and uptake of digital health solutions such as telemedicine [3,4], including the use of remote digital dermatology care [5-7]. Digital dermatology allows general practitioners (GPs), the patient’s first point of contact, to digitally contact the patient or to consult a remote dermatologist for advice [8-11]. Digital dermatology is suitable for web-based assessment of skin lesions because it provides a digital representation of the skin. Moreover, this type of service has enabled Dutch GPs to continually provide dermatology care to patients while minimizing the number of (unnecessary) conventional face-to-face consultations (in dermatology or GP practice) and the risk of exposure to SARS-CoV-2.

The Netherlands is one of the few countries where an integrated remote digital dermatology service in GP care has been operating, integrated, and fully reimbursed since 2006 [12]. Therefore, it was expected that GPs could smoothly apply the service in their work practices during the COVID-19 pandemic. However, how the pandemic subsequently influenced the existing digital dermatology care delivery and affected the Dutch GP work processes remains unknown.

The digital dermatology service cannot be adequately evaluated in isolation from the organizational context in which it is implemented. Organizational factors such as the lack of adequate training and technological support, existing and new policies, leadership and change management, and communication needs can hinder the adoption and implementation of digital health tools [13,14]. Moreover, digital dermatology is used in a complex health system that consists of numerous interconnected components (eg, technological elements and social human system aspects) that interact and must work together to positively contribute to the delivery of such a service [15,16]. Digital services affect the work processes of health care providers and the way in which they deliver care to patients. Ideally, such telemedicine service should be seamlessly incorporated into the provider’s day-to-day work processes [13], but achieving that goal requires insight into the aspects that affect GPs’ satisfaction and the continued use of the service.

Sociotechnical models provide a framework to focus on a broad range of factors that influence the use and adoption of health IT and incorporate technical and nontechnical factors [17]. In other words, the entire implementation process and evaluation of a digital innovation includes the interactions among the technical, social, workflow, and organizational factors. These factors are closely interrelated and are crucial for understanding the complex picture of health care innovations [18].

### Objective

To evaluate health care providers’ experiences with store-and-forward telemedicine services from a contracted telemedicine organization perspective and to assure telemedicine service quality, we previously developed and validated the Store-and-Forward Telemedicine Service User-satisfaction Questionnaire (SAF-TSUQ) [19]. However, this questionnaire does not focus on the interrelations between aspects from a broad sociotechnical perspective. A sociotechnical framework can be used to enhance the analysis of open-ended questions and to model the interrelated aspects of digital dermatology care. In addition, such a framework can be used to identify the sociotechnical facilitators and barriers that influenced GPs regarding the use of remote digital dermatology care during the pandemic. These findings on GPs’ perspectives about digital dermatology care can be used to support future sustainable use of this service in daily practice.

Therefore, in this study, the following research questions were answered:

1. How do Dutch GPs experience the remote dermatology care service quality, based on the SAF-TSUQ questionnaire from a contracted telemedicine organization perspective?
2. Which facilitators and barriers do GPs experience in remote dermatology care from a sociotechnical perspective?

## Methods

### Overview

**Figure 1:**
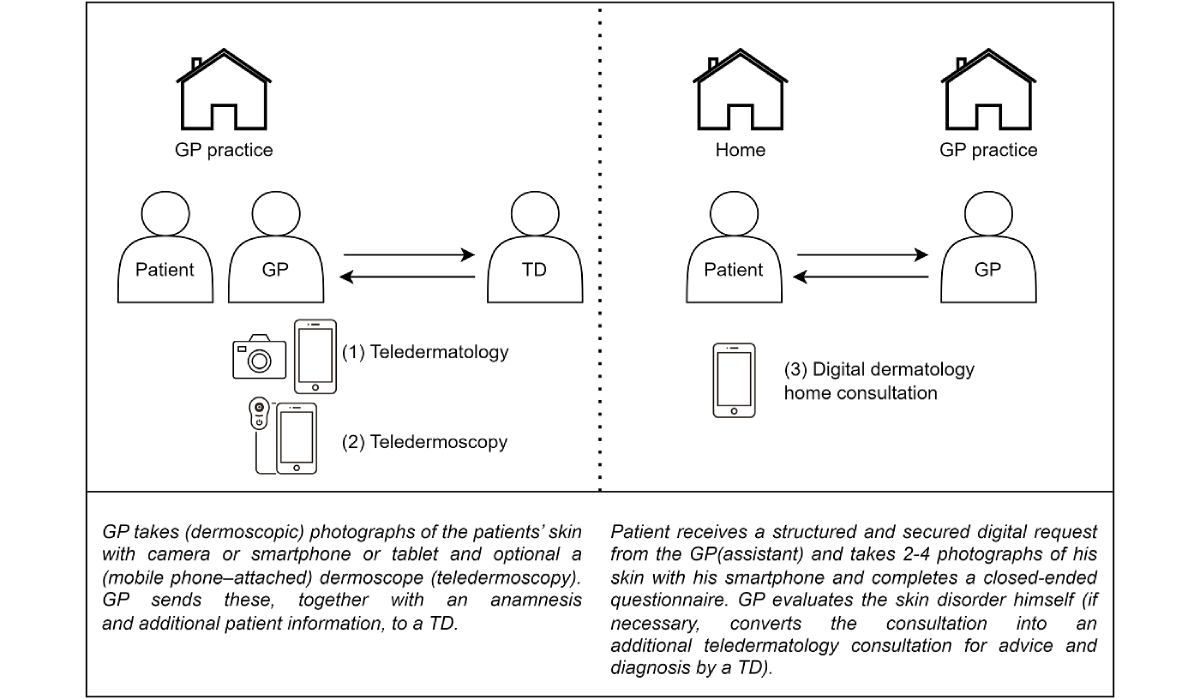
Teledermatology, teledermoscopy, and digital dermatology home consultation process. GP: general practitioner; TD: teledermatologist.

Ksyos [20] is one of the largest health care organizations in the Netherlands that facilitates three types of remote store-and-forward digital dermatology care: (1) teledermatology, (2) teledermoscopy, and (3) dermatology home consultation (Figure 1). Ksyos-affiliated health care providers acknowledge and approve that Ksyos monitors the quality of its telemedicine services and conducts scientific research when they register for an account on the Ksyos telemedicine platform. The Amsterdam University Medical Center (location: Academic Medical Center) performed this study in collaboration with Ksyos. Data collection for this study was conducted between December 2021 and March 2022 by a researcher (ET).

### Ethical Considerations

The Medical Ethical Commission of the Amsterdam University Medical Center granted a waiver stating that the study did not require additional approval.

### Participants

Ksyos invited all affiliated GPs (approximately 3257 GPs) to complete the questionnaire. These GPs were able to perform a store-and-forward digital dermatology consultation and had started a digital consultation request (not necessarily dermatology) between October 2019 and September 2021, and their email addresses were known. Owing to outdated email addresses or accounts, we were unable to determine the exact number of invited GPs.

### Procedure

The questionnaire invitation email included a personalized URL link to an anonymous web-based questionnaire tool called LimeSurvey. The URL link was deactivated when the questionnaire was completed, to prevent multiple participation. It was impossible to link this URL to the provided responses. Owing to technical issues in the email tool, it was impossible to send multiple invitation emails to GPs registered with the same general GP practice email address. If this was the case, the email tool chose only 1 recipient.

After 1 week, nonresponding GPs received a reminder email. Participation was voluntary, and GPs could unsubscribe via email. In total, 4 gift cards worth €50 (US $53.65) were raffled among all responding health care providers in a large study.

### Questionnaire Instrument

**Figure 2:**
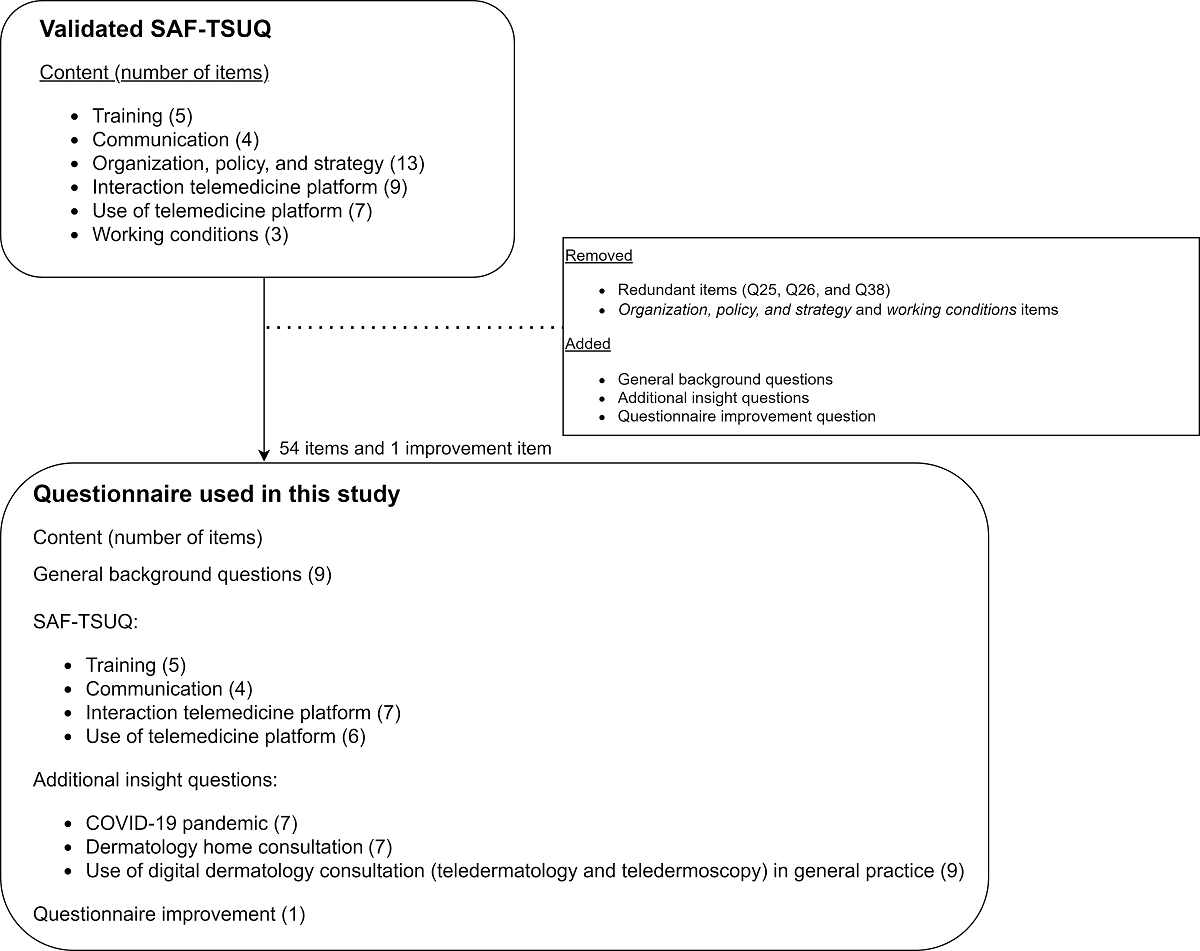
Questionnaire instrument. General background questions: for example age, sex, the frequency of telemedicine platform use, years of experience with telemedicine platform, self-reported computer skills, and technology adoption. COVID-19 pandemic questions: the frequency of telemedicine platform use, experiences and lessons learned with teledermatology, teledermoscopy, and digital dermatology home consultation. Dermatology home consultation questions: general practitioners’ (GPs) experiences with digital dermatology home consultation (ie, image quality with patients as photographers and needed improvement, which skin conditions and population are suitable for digital dermatology home consultation, and other patient-GP delivery modalities used). The use of digital dermatology consultation in general practice questions: reasons for (not) performing a digital dermatology consultation, the photographer of the (dermoscopic) photographs, the dermatologist feedback received about the quality of the photographs, suggested improvements to optimize photograph quality, GPs’ confidence in teledermatology and teledermoscopy use, and the extent of image training received. SAF-TSUQ: Store-and-Forward Telemedicine Service User-satisfaction Questionnaire.

The web-based GP questionnaire (Multimedia Appendix 1) was available in Dutch only and consisted of 54 open-ended and closed-ended questions. The questionnaire included general background questions, questions from the validated SAF-TSUQ questionnaire [19], and newly added insight questions related to the pandemic and use of digital dermatology care in general practice (Figure 2). The SAF-TSUQ questions evaluated the service quality as experienced by GPs from a contracted telemedicine organization perspective, whereas in-depth insight questions were added to evaluate digital dermatology care as experienced by GPs from a broad sociotechnical perspective.

Answers to the SAF-TSUQ questions were recorded on a 5-point Likert scale (range: 1=strongly disagree to 5=strongly agree) and the nonsubstantive options, “I do not know” and “not applicable.” Overall, 3 redundant items of the original SAF-TSUQ were discussed with a quality manager at Ksyos and were removed beforehand. In addition, the questions related to “organization, policy, and strategy” and “working conditions” were excluded because during the validation of the original SAF-TSUQ questionnaire among all health care providers, the Dutch GPs frequently reported that these questions were not applicable to them in the Dutch context. Furthermore, the newly added questions related to the pandemic were formulated based on questions that emerged out of interest from the researchers during the COVID-19 pandemic. The other additional in-depth insight questions were related to the use of teledermatology, teledermoscopy, and digital dermatology home consultation in general practice (Figure 2). These questions were also specifically formulated for this study and focused on training and image quality, as these factors are often mentioned as barriers to telemedicine use [21-23].

For some of the closed-ended questions, GPs were prompted in an open-ended follow-up question to explain why they chose a specific answer category. At the end of each section, a separate textbox was presented for additional free-text comments. Furthermore, 1 final open-ended feedback question was included to gather any feedback or suggestions from GPs for improvement of the questionnaire. All questions (except the additional free-text comments and open-ended follow-up questions) were mandatory. A GP resident and a GP reviewed the newly added questions and options in advance. Then, 2 researchers (ET and Femke van Sinderen) evaluated the questionnaire’s technical operation, and a data management consultant (Miranda Roskam-Mul) externally reviewed the questionnaire’s technical operation.

### Data Analysis

This study excluded the responses of GPs who responded that they did not use digital dermatology consultation in the Ksyos platform and did not consent in the questionnaire to anonymously process their answers for scientific purposes. Furthermore, incomplete questionnaires, vague or incomprehensible free-text or open-ended responses, and comments that related to a care path other than dermatology were excluded from data analysis. Incomplete questionnaires were excluded, as this was the only way to prevent the inclusion of questionnaires submitted by the same GP.

Descriptive statistics were used to analyze the single-choice, multiple-choice, and Likert scale responses, using numbers and percentages (R software, version 4.0.3; R Foundation for Statistical Computing) [24].

Overall, 2 researchers (ET and Bibiche Groenhuijzen) independently read all open-ended and free-text responses line by line and applied *(thematic)* content analysis to get a deep understanding of the issues GPs experience when using remote digital dermatology care. The sociotechnical model was developed by Sittig and Singh [25] to identify the sociotechnical issues that arise during the design, development, implementation, use, and evaluation of health IT within complex health care systems. We applied their model to group the open and free-text responses into 8 interrelated dimensions: (1) hardware and software; (2) clinical content; (3) human-computer interface; (4) people; (5) workflow and communication; (6) internal organizational policies, procedures, and culture; (7) external rules, regulations, and pressures; and (8) system measurement and monitoring.

During axial coding, 1 coder (Bibiche Groenhuijzen) applied a subcode for each answer, and main codes were formulated (LWP and Bibiche Groenhuijzen) and assigned to each subcode. Most answers were short; however, some answers contained more detailed information and were assigned to multiple codes. The codes were applied to categorize the open-ended responses systematically and to compare the data with other similar parts of the data set. The second coder (ET), a Ksyos expert, assigned a subcode and main code to each response and, if necessary, added additional subcodes. After coding the first few responses, an informative meeting between the researchers (ET and Bibiche Groenhuijzen) was conducted to discuss how coding proceeded till then and any uncertainties about the process and definitions of the codes. Then, the second coder (ET) finalized the coding. This second coder had access to the list of predefined subcodes and main codes but was blinded to the previous codes assigned to free-text and open-ended responses by the first coder. Both researchers (ET and Bibiche roenhuijzen) classified the responses as facilitating, impeding the use of digital care, or neutral and assigned a sociotechnical dimension of the 8-dimensional model by Sittig and Singh [25] to the responses.

Finally, responses that were coded differently or assigned to a different dimension were discussed until consensus was reached (ET and Bibiche Groenhuijzen). Several iterations were conducted to reach consensus in assigning codes to responses and to modify the descriptions of the original 8 sociotechnical dimensions in the telemedicine context (Table 1). Finally, based on this complete analysis, we extracted the facilitators of and barriers to digital dermatology care from the 8 sociotechnical dimensions.

**Table 1:** Definitions based on the 8-dimensional sociotechnical model by Sittig and Singh modified to the telemedicine context [25,26].

Sociotechnical dimension	Definition in the model
Hardware and software	All technical remarks about the hardware and software used on the (teledermatology) consultation platform, for example, the ease of use of the photography equipment, uploading images, and interoperability issues.
Clinical content	All remarks about the structured, unstructured, textual, or numeric data; information; and knowledge that are stored on the (teledermatology) consultation platform. Also remarks about (the feedback received from the dermatologist about) the quality of images in the consultation or the quality of responses of the dermatologist.
Human-computer interface	All remarks about the software’s interaction with the user, for example, about the platform layout or front-end features.
People	All remarks about individuals who interact with the platform or related to training and learnability.
Workflow and communication	All remarks about how teledermatology is used in the workflow, impact on workload, tasks required to provide appropriate care, and communication with the telemedicine organization.
Internal organizational policies, procedures, and culture	All remarks about structures, policies, financial aspects, and procedures of the telemedicine organization that influence technology management.
External rules, regulations, and pressures	All remarks about external forces outside the telemedicine organization that facilitate or impede efforts to design, implement, use, and evaluate technology and remarks indicating that the use has changed owing to the COVID-19 pandemic.
System measurement and monitoring	All remarks about platform availability, its use by stakeholders, its effectiveness, and associated intended and unintended consequences. This dimension also includes comments in which participants indicate that the COVID-19 pandemic had no effect.
Not able to code	All remarks that were not sufficiently specific or not comprehensive to be assigned to a dimension. Remarks about the questionnaire itself are also included in this dimension.

## Results

### Participant Characteristics

Of 3257 GPs, 40 (1.23%) GPs were retired, no longer worked in the GP practice, no longer used their email address, were absent for a long time, or unsubscribed themselves from the study. Overall, 71 GPs indicated performing digital dermatology consultations and completed the entire questionnaire. If all these remaining 3217 GPs received and read the email, this would indicate a response rate of 2.21% (71/3217); however, it is possible that several emails were not delivered or read; therefore, the response rate could not be determined and might be underestimated.

Of the 71 GPs, 5 (7%) did not provide consent for the use of their data for scientific purposes and were therefore excluded. Table 2 presents the background characteristics of the remaining 93% (66/71) of the GPs. Of the 66 GPs, most GPs were female (n=36, 55%), aged between 35 and 44 years (n=25, 38%), weekly platform users (n=33, 50%), working with the telemedicine organization for >5 years (n=34, 52%), and reported themselves as early majority adopters (n=44, 67%) with good computer skills (n=30, 45%).

**Table 2:** Background characteristics of the responding GPs^a^ (n=66).

Characteristics			GPs, n (%)
**Age range (years)**			
	18-24	0 (0)	
	25-34	2 (3)	
	35-44	25 (38)	
	45-54	17 (26)	
	55-64	19 (29)	
	≥65	3 (5)	
**Sex**			
	Male	30 (45)	
	Female	36 (55)	
**Frequency of use**			
	Daily	10 (15)	
	Weekly	33 (50)	
	Monthly	19 (29)	
	A few times in a year	4 (6)	
	Never	0 (0)	
**Working with telemedicine organization**			
	<6 months	0 (0)	
	6-12 months	1 (2)	
	1-3 years	14 (21)	
	3-5 years	17 (26)	
	5-10 years	22 (33)	
	>10 years	12 (18)	
**Use of other telemedicine care pathways^b^**			
	Cardiology	38 (58)	
	Laboratory requests	5 (8)	
	Mental health	15 (23)	
	Ophthalmology	31 (47)	
	Pulmonology	6 (9)	
	Sleep	30 (45)	
**Self-reported computer skills**			
	Poor	2 (3)	
	Sufficient	20 (30)	
	Good	30 (45)	
	Excellent	14 (21)	
**Adopter category**			
	Innovators	3 (5)	
	Early adopters	9 (14)	
	Early majority	44 (67)	
	Late majority	10 (15)	
	Laggards	0 (0)	

^a^GP: general practitioner.

^b^The total response percentage exceeds 100% because multiple responses were allowed.

### Responses to SAF-TSUQ

**Figure 3:**
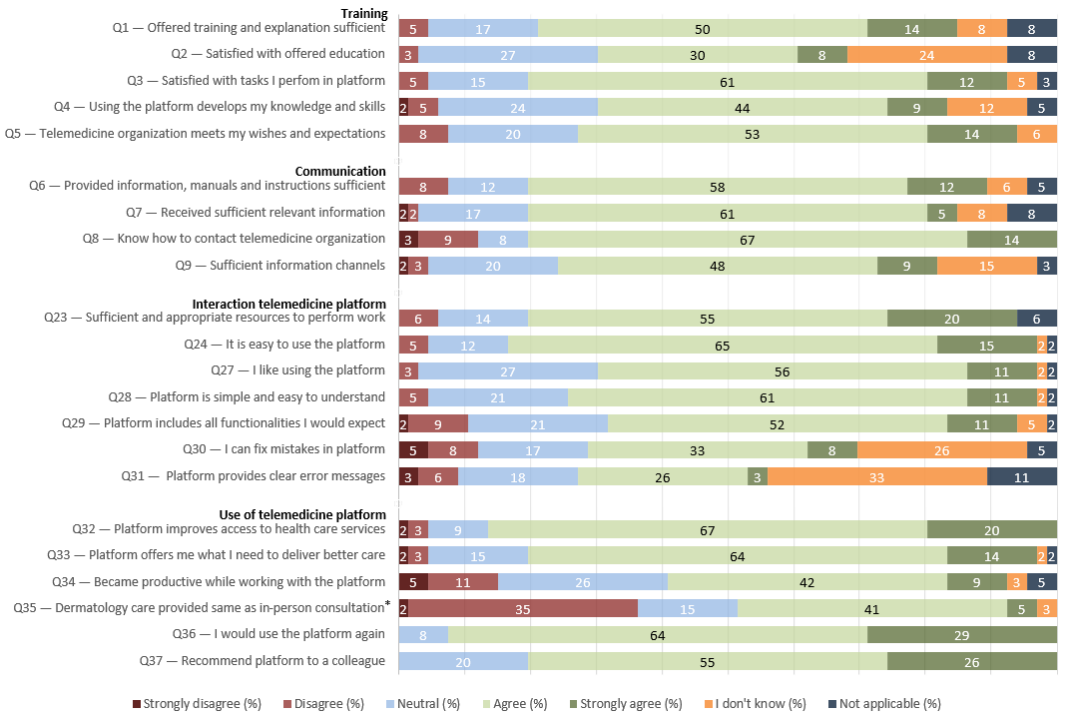
Store-and-Forward Telemedicine Service User-satisfaction Questionnaire responses to the training, communication, interaction, and use statements. *Additional explanation in the questionnaire—by this question, we meant that a digital consultation can replace a regular consultation.

The responding GPs were positive about training, communication, the use of, and interaction with the telemedicine platform (Figure 3). Almost all GPs would use the platform again (61/66, 92%) and recommend it to a colleague (53/66, 80%). Most GPs (42/66, 64%) found the training and explanation offered by the telemedicine organization as sufficient to be able to use the platform in their daily practice. However, one-third (21/66, 32%) of the GPs were not familiar with the options for additional or continuing education offered by the telemedicine organization. Overall, the results of the SAF-TSUQ show opportunities for improvement regarding interaction with the platform (eg, missing functionalities or the lack of knowledge about how to rectify or avoid mistakes). Notably, more than one-third (24/66, 36%) of GPs disagreed that the digital dermatology care provided with the telemedicine platform can be considered as a replacement of an in-person dermatology consultation.

### Responses to Additional Insight Questions

Closed-ended responses to the additional insight questions are presented in Table 3. Most GPs (42/66, 64%) reported that they used the telemedicine platform approximately as often during the first COVID-19 wave as in the period before the COVID-19 pandemic, whereas 23% (15/66) of the GPs used the telemedicine platform more often. Of the 66 GPs, 46 (70%) GPs used the telemedicine platform at the time of this study as often as before the pandemic, and 16 (24%) GPs used the platform more frequently. Of the 66 GPs, 40 (61%) had (strongly) positive experiences with the use of digital dermatology during the pandemic. Almost all GPs (60/66, 91%) received sufficient support to provide digital care. Of the 66 GPs, 63 (95%) took the (dermoscopic) photographs during a teledermatology or teledermoscopy consultation themselves. Of the 66 GPs, only 30 (45%) received training or instructions about taking (dermoscopic) photographs from the telemedicine organization, 7 (11%) took an (additional) imaging course (eg, dermoscopy and medical imaging), and 13 (20%) gained experience in taking (dermoscopic) images in daily practice. Of the 66 GPs, 25 (38%) GPs did not receive any training or instruction, and 15 (60%) of them did not judge this training or instruction as necessary. Of the 66 GPs, 60 (91%) felt (strongly) confident and 5 (8%) felt “neutral” confident in determining the patient’s treatment policy after a teledermatology or teledermoscopy consultation (based on the advice and diagnosis received from the dermatologist). GPs primarily use digital dermatology consultations to prevent physical referrals of their patients to a dermatologist (54/66, 82%).

**Table 3:** GPs’^a^ responses to additional insight questions (n=66).

Questions and response options			GPs, n (%)
**Platform use during the first COVID-19 wave^b^ compared with before the COVID-19 pandemic**			
	Less often	9 (14)	
	Approximately as often	42 (64)	
	More often	15 (23)	
	Not applicable	0 (0)	
**Current^c^ platform use compared with that during the period before the COVID-19 pandemic**			
	Less often	3 (5)	
	Approximately as often	46 (70)	
	More often	16 (24)	
	Not applicable	1 (2)	
**Received sufficient support to provide digital care during the COVID-19 pandemic**			
	Yes	60 (91)	
	No	6 (9)	
**Experiences regarding consultations in digital dermatology care pathway during the COVID-19 pandemic**			
	Strongly negative	0 (0)	
	Negative	3 (5)	
	Neutral	18 (27)	
	Positive	32 (48)	
	Strongly positive	8 (12)	
	Not applicable	5 (8)	
**Reasons for using digital dermatology in daily practice^d^**			
	Preventing physical referrals	54 (82)	
	Unable to determine a differential diagnosis	46 (70)	
	Treatment is unsuccessful	41 (62)	
	Receiving additional advice	40 (61)	
	Lower costs for the patient	27 (41)	
	Long waiting times in hospitals	21 (32)	
	At the request of the patient	8 (12)	
	Suspicion of malignancy	3 (5)	
	Doubts about the size of the deviation	3 (5)	
	Emergencies	1 (2)	
	Prevent physical consultation in practice owing to crowds or SARS-CoV-2 infection of a patient	1 (2)	
**Used digital dermatology home consultation**			
	Yes	10 (15)	
	No	56 (85)	
**Experiences regarding digital dermatology home consultation (n=10)**			
	Strongly negative	0 (0)	
	Negative	1 (10)	
	Neutral	1 (10)	
	Positive	7 (70)	
	Strongly positive	1 (10)	
**Quality of the photographs taken by patients (n=10)**			
	Always poor	0 (0)	
	Usually poor	1 (10)	
	Sometimes good, sometimes poor	5 (50)	
	Usually good	4 (40)	
	Always good	0 (0)	
**Age preference for digital dermatology home consultation (n=10)**			
	No age preference at all	6 (60)	
	Solely for babies	1 (10)	
	Solely for children aged <12 years	1 (10)	
	For babies, toddlers, and adults	1 (10)	
	Any patient with a smartphone	1 (10)	

^a^GP: general practitioner.

^b^The first COVID-19 wave was defined as the start of the pandemic (March 2020 to May 2020).

^c^At the time of this study (December 2021 to March 2022).

^d^The total response percentage exceeds 100% because multiple responses were allowed.

Of the 66 GPs, 10 (15%) used digital dermatology home consultation and 80% (8/10) of them were (strongly) positive about their experiences. These GPs perceived digital dermatology home consultation as specifically suitable for skin conditions with red discoloration (10/10, 100%), birthmarks (3/10, 30%), bumps (7/10, 70%), wounds (7/10, 70%), and diaper rash (8/10, 80%). GPs had no evident age preference for which patients digital dermatology home consultation is the most appropriate. In addition, GPs reported divergent experiences with the quality of photographs taken by patients.

### Qualitative Analysis of Free-Text and Open-Ended Responses

#### Overview

The 66 GPs provided a total of 385 answers to the open-ended questions. Furthermore, they provided 100 and 35 additional free-text answers in the separate textboxes at the end of each section and the last questionnaire improvement question, respectively. After the exclusion of the no responses (116/520, 22.3%) and not applicable (dermatology) responses (22/520, 4.2%), a total of 324 responses to the open-ended questions and 58 free-text responses remained. Overall, 12.3% (47/382) of the remaining responses contained additional information, and after splitting these into 2 or 3 responses, this resulted in 436 remarks for the qualitative data analysis. Then, 2 researchers (ET and Bibiche Groenhuijzen) mapped these remarks across all 8 sociotechnical dimensions. No third reviewer was needed to reach an agreement between the 2 raters. Most remarks (97/413, 23.5%) were assigned to the *clinical content* dimension*,* followed by *system measurement and monitoring, internal organizational policies, procedures, and culture,* and *people* (Table 4).

**Table 4:** Exemplary quotes of open-ended and free-text remarks for each of the 8 dimensions of the sociotechnical model by Sittig and Singh [25].

Dimension	Facilitating quotes (%)	Neutral quotes (%)	Impeding quotes (%)	Exemplary relevant quotes
Hardware and software (n=38)	2 (5)	2 (5)	34 (89)	“The conversion lens on the phone for dermoscopic photographs does not meet the quality of the photographs that we had previously with the dermoscope. I often hear from dermatologists that they cannot interpret the photographs properly and patients still have to go to the specialist.” [ID 1625]^a^ “Uploading photographs [from my phone to my desktop] takes a lot of time and often has to be done again because the amount of MBs is exceeded.” [ID 1376]^a^ “Teledermoscopy remains difficult, ‘every phone [requires] a new [dermoscopy] attachment.’” [ID 1259]^a^
Clinical content (n=97)	24 (25)	42 (43)	31 (32)	“Many skin problems, spots, and rashes can often be easily assessed via [a] photograph.” [IDs 1637 and 1804]^b^ “Fast responses, good content and practical responses from specialists with clear and adequate diagnoses and treatment advice.” [IDs 109, 1373, 1479, and 1344]^b^ “Images [taken by patients] are often not sharp enough, bad lightning, wrong distance.” [DHC^c^; ID 1638]^a^ “I find the quality and sharpness of the patient’s photographs extremely poor. For example, [there is] only a detailed image or [the image is] too out of focus. I prefer to take my own photographs and send them in with an adequate anamnesis.” [DHC; ID 1373]^a^
Human-computer interface (n=16)	1 (6)	3 (19)	12 (75)	“Only allow patients to send in a [dermatology home] consultation when all photographs have been taken and loaded.” [DHC; ID 1691]^d^ “Less change in well-intentioned updating of layout.” [ID 1660]^a^ “The patient found it [dermatology home consultation] extremely difficult and user unfriendly.” [DHC; ID 1692]^a^
People (n=65)	8 (12)	51 (78)	6 (9)	“[I learned] which questions are suitable for teleconsultation.” [ID 1710]^b^ “The learning capacity [of digital dermatology consultation] is strong. I notice that the number of consultations has decreased, partly due to the learning curve of comparable consultations.” [ID 1692]^b^ “The patient’s photography skills are on average poor (and so are the photographs).” [DHC; ID 1257]^a^ “The patient is not trained how [to take photographs].” [DHC; ID 1692]^a^
Workflow and communication (n=13)	3 (23)	4 (31)	6 (46)	“Patients like [digital consultations] for a while but then want to be seen [physically] again. However, there is an increase in e-consultations that is partly extra and possibly better care, but certainly no relief from work.” [ID 1188]^a^
Internal organizational policies, procedures, and culture (n=78)	1 (1)	66 (85)	11 (14)	“[I take photographs] with my own camera on [my] smartphone. That [camera] is fine. Only the attachment no longer fits and is no longer supplied unfortunately. Another stand-alone USB version would be possible, but it costs quite a lot. A little discount through [the telemedicine organization] (win-win) would have been nice...It is a pity that [the telemedicine organization] does not invest so much in teledermatology anymore. Devices used to be ‘free’ if you performed enough [consultations]. Now that is no longer the case, I perform fewer [consultations]. Because the attachment no longer fits. I think that we both benefit less from that. Then I just refer to the dermatologist.” [ID 1363]^a^ “Offer better phones as was common practice 4 years ago. This is an extra incentive to use the dermatology service and guarantees quality of the photo cameras.” [ID 1349]^a^
External rules, regulations, and pressures (n=21)	11 (52)	5 (24)	5 (24)	“Digital care where I [as GP] seek secondary care [dermatologist] for advice has not been increased. Digital care where I was able to come to a solution together with the patient has been increased.” [ID 1692]^b^ “[We] already used teledermatology and teledermoscopy, but [we] have also started to digitally assess [patients’] skin conditions ourselves.” [ID 1540]^b^ “Before [the pandemic] we did not allow patients to send in pictures; that has naturally crept in during the COVID-19 pandemic; this with a satisfying result.” [DHC; ID 1366]^b^ “Digital dermatology was already an absolute winner before the COVID-19 pandemic. [Digital dermatology was used] less [often] during [the] COVID-19 [pandemic] because patients [were] preferably not [seen] live in [GP] practice, so spots had to wait.” [ID 1257]^d^ “[Digital dermatology] can lead to risky contacts at less than 1.5 meters.” [ID 1257]^a^
System measurement and monitoring (n=85)	56 (66)	27 (32)	2 (2)	“Quick and easy for the patient, without [physical] referral with a long waiting time.” [ID 1667]^b^ “[Digital dermatology consultations are] a great way to communicate with [a] dermatologist.” [ID 1362]^b^ “It is nice to have [the patient] observed remotely and to be able to keep the patient out of the hospital.” [IDs 1313 and 1710]^b^
Not able to code (n=23)	N/A^e^	N/A	N/A	N/A

^a^Impeding quote.

^b^Facilitating quote.

^c^DHC: digital dermatology home consultation.

^d^Neutral quote.

^e^N/A: not applicable.

#### Facilitators

During the pandemic, GPs found digital dermatology care to be reliable, fast, and time efficient (accelerates care delivery). GPs experienced substantive good, practical, and fast responses from the specialists, including adequate diagnoses and treatment recommendations for their patients with skin lesions. GPs found it positive that they themselves remained responsible for the care of their patients. Overall, 26% (17/66) of the GPs indicated that digital care and digital dermatology consultations (partly) replaced physical consultations of their patients in primary and secondary care. A GP reported that he temporarily requested a few more teleconsultations in dermatology during the pandemic, but this number dropped after some time. GPs expressed that they learned from the feedback provided by teledermatologist and for which patient symptoms a teleconsultation is beneficial. Furthermore, a GP reported that the number of digital dermatology consultations that he requested to a dermatologist has decreased because he learned from the feedback from similar previous consultations.

#### Barriers

##### Limited Digital Photography Skills of Patients and GPs

The first barrier that GPs encountered relates to the limited digital photography skills of GPs and their patients. GPs reported to receive poor or nonassessable photographs from their patients because their patients have poor photography skills as they are not specifically trained in how to take photographs of their skin lesion. More specifically, GPs reported that patients provided skin photographs that were not sharp (when zoomed in), lacked proper details, were blurry or had poor lighting, were taken from a wrong distance, and did not always have good shades of color. In particular, overview, detailed, and magnified photographs of patients’ skin lesions were not optimal, or patients provided an insufficient number of photographs. GPs reported that good web-based support and an understandable guide are needed to ensure good quality of photographs taken by patients; otherwise, their ignorance about how to take pictures will lead to many additional, time-consuming questions from patients to the GP. GPs expressed the need for a quality warning system if images uploaded by a patient are incomplete and not with sufficient quality.

Furthermore, approximately half (35/66, 53%) of the GPs indicated that they received complimenting or constructive feedback from a dermatologist about the quality of their photographs. Although most GPs (42/66, 64%) were positive about the provided training, they suggested additional (web-based) training options such as short video instructions as refreshers, practice sessions about photographing skin lesions with their own (dermoscopy) equipment, and advice about using the mobile phone camera. Others do not consider training as necessary to use the service.

##### Lack of Appropriate Up-to-Date Imaging Equipment and Equipment Costs

The second barrier relates to the lack of up-to-date, appropriate, digital dermatology imaging equipment and equipment costs. In the past, the telemedicine organization offered up-to-date, free-of-charge equipment to GPs in exchange for performing a minimum number of digital dermatology consultations, but they do not provide this equipment anymore. Nowadays, technology develops rapidly and GPs must purchase the latest off-the-shelf equipment themselves. GPs reported that especially dermoscopes are very expensive and that the provision of digital dermatology imaging equipment by the telemedicine organization is an extra incentive to use the service. GPs missed an appropriate mobile phone–attached dermoscope or had troubles with using the outdated attachment and reported that their photographs were not with sufficient quality with the current conversion lens.

##### Human-Computer Interface and Interoperability Issues

The third barrier relates to the human-computer interface and interoperability issues on the telemedicine platform. Interface issues included platform usability issues, strict validation on capitalization of address data, linking new user accounts, and changing layout. Interoperability issues included difficulties in uploading all patient information (eg, medical history, medication, and address) from the GP Information System (*Dutch: Huisarts Informatie Systeem*) into the digital dermatology consultation and vice versa in loading relevant patient data back from the digital consultation into the GP Information System. Furthermore, for teledermatology and teledermoscopy, most GPs take images with their mobile phones and upload these images via an app into the digital dermatology consultation form. Subsequently, on their computer, they complement the digital dermatology consultation form and send it to a teledermatologist. This process is time-consuming and complex; therefore, GPs prefer to start the digital dermatology consultation on their phones and directly send the consultation request and the images from their phone to a dermatologist.

##### Different Use Procedures

The fourth encountered barrier is that GPs have various reasons to use or to not use the service. Of the 66 GPs, 5 (8%) GPs reported no threshold for use at all, whereas other GPs experience thresholds for use. For example, if a patient has >1 skin abnormality, they have to create a new consultation for each abnormality. Another threshold for use is if they are not able to upload the images. Other reasons for a GP to not request a digital dermatology consultation are when an in-person visit or treatment or biopsy in the hospital is required anyway, or when in their opinion, a digital dermatology consultation is not indicated. In addition, GPs do not perform teledermatology when the patient prefers a physical consultation or disagrees with a digital consultation, for atypical or pigmented nevi for which skin inspection by touch is required for a correct diagnosis, for unclear skin abnormalities, for common skin lesions, or for urgent skin problems such as suspicion of melanoma or malignancy (36/66, 55%). In the latter possibly malignant cases, GPs perform a biopsy themselves or refer the patient to a dermatologist. However, 6% (4/66) of the GPs indicated to apply digital dermatology care for emergencies and lesions that are suspected to be malignant.

### Questionnaire Improvement

Only 4 suggestions for improving the questionnaire were given and 2 support questions were asked. The remaining GPs had no comments or were satisfied with the questionnaire.

## Discussion

### Principal Findings

Overall, GPs had positive experiences with remote digital dermatology care during the COVID-19 pandemic. However, despite these positive perspectives, important barriers of the digital dermatology service were revealed regarding GPs' and patients’ limited digital photography skills, costs and the lack of appropriate imaging equipment, human-computer interface and interoperability issues, and different use procedures.

### Comparison With Previous Studies

Most GPs (46/66, 70%) in our study used the telemedicine platform approximately as often at the time of this study as before the pandemic. In contrast, the Netherlands Institute for Health Services Research (*Dutch: Nederlands instituut voor onderzoek van de gezondheidszorg* [NIVEL]) reported that 52% of Dutch GP practices intensified their teleconsultation contacts with medical specialists during the first COVID-19 wave, but GPs from these practices considered this only as a slight increase in teleconsultation use [27,28]. Furthermore, studies in other countries showed that dermatologists saw an increase in the number of remote dermatology consultations that they assessed during the pandemic in comparison with that during the prepandemic period [29,30]. Possible reasons for this lack of growth in teleconsultations requested by GPs in our study were, first, the service was already successfully implemented before the pandemic and, second, during the pandemic, patients were not only avoiding hospital care but also GP care. Teledermatology and teledermoscopy had the potential to reduce the number of physical referrals to hospitals but also required the patients to visit the GP’s practice with possible physical contact at <1.5 m (4.9 feet). Patients were still hesitant to physically contact a GP because of the risk of exposure to the virus [1,31]. As a complementary service to the conventional face-to-face dermatology consultation in GP practice, digital dermatology home consultation, which was already in practice, took off. With this service, patients could take the photographs themselves with their own mobile phone or smartphone device and send these photographs securely to the GP for assessment without waiting time, physical contact, and the risk of contamination in GP practice. Therefore, a new group of complaints related to skin disorders that were normally handled physically by the GPs in their practice was submitted digitally by the patient. This meant that the pandemic had changed the spectrum of skin disorders managed and the profile of patients. Digital dermatology consultation was no longer used by GPs solely for difficult-to-assess skin complaints but also for easy-to-assess skin complaints sent in by patients that were usually assessed in GP practice. However, digital dermatology home consultation requires that patients have the appropriate equipment and technical literacy to engage the service on their own. Despite that most patients had access to a mobile phone or smartphone [32], they were not trained to take photographs of their skin condition. Therefore, GPs in our study reported images of mostly inadequate quality taken by patients. This shows that the external pressure of the pandemic pushed the use of remote dermatology care by (new and untrained) people; however, the fact that these users had insufficient knowledge about the requirements for taking appropriate photographs led to problems with the clinical assessment of the photographs. Future studies could investigate whether the skin disorders in the remote store-and-forward digital dermatology care population changed in comparison with the prepandemic period.

A Spanish study during the pandemic confirmed that patients had limited photography skills [33]. They found that only half (52.1%) of the images captured by patients and directly sent to the dermatologist were of adequate quality. Furthermore, in approximately one-fourth of these cases, poor image quality of these patient-submitted images was the reason why the teledermatologist could not provide a diagnosis. A prepandemic American study showed that a slightly higher percentage (62.2%) of the images sent by a patient to a dermatologist via teledermatology were with sufficient quality, whereas dermatologists perceived only half of the total images as having sufficient quality for decision-making [34].

Besides the remarks of GPs in our study about the photography skills of the patients, GPs also reported that they received constructive or complimentary feedback from dermatologists about the quality of their photographs. Previous studies about image quality of photographs taken in primary care for digital dermatology consultation also have demonstrated diverse results [10,35-39]. Poor photograph quality in these studies was, similar to our study, caused by out-of-focus images or missing overview or dermoscopic images of a patient’s skin lesions. Digital dermatology consultations can be performed using current technologies, but many of the pictures are of unacceptable quality, and the training of health care providers and patients in taking images should thus be considered [21]. In the Dutch GP training curriculum, GPs are, in general, not trained to use digital services [40]. Only 5% (3/66) of GPs in our study indicated that they received training for taking (dermoscopic) photographs in their GP training curriculum. Owing to this lack of training in the GP curriculum, the telemedicine organization (Ksyos) organizes personal training sessions about the use of the digital dermatology service for all newly operating GP practices. Despite this introductory training, only about half (30/66, 45%) of the GPs in our study reported that they opted for this training or instruction about taking (dermoscopic) photographs from the telemedicine organization. This indicates that GPs did not experience this introductory training as an official education or instruction moment but solely as an installation or demonstration. In addition, a few GPs in our study took a (follow-up) imaging course. Although most GPs were satisfied with the training they received or indicated that they felt no need for (additional) training, many photograph quality issues were revealed in our study. This shows that training of people influences the quality of the images captured during a remote dermatology consultation. Furthermore, more than one-third (25/66, 38%) of the GPs used the telemedicine platform monthly or only a few times in a year. Such a long interval between uses might dilute their acquired skills and knowledge [41]. Therefore, our results suggest that GPs need continuous web-based and good practice training sessions and video instructions as refreshers (eg, instructions to refresh their knowledge about the use of the equipment to capture images and how to use the platform). We propose that these training sessions are accredited by the European Accreditation Council for Continuing Medical Education, which might stimulate GPs across Europe to participate in training sessions and to use digital dermatology services [42]. Furthermore, (video) instructions about how to obtain adequate photographs and an understandable, straightforward, step-by-step (web-based) guide for GPs and patients should be provided by the telemedicine organization to mitigate the image quality barrier in the future.

In addition, our results showed that the quality of the (dermoscopic) images was not only dependent on the photography skills of the patients and GPs but also on the imaging devices used in daily practice. These equipment issues related, among others, to the (outdated) mobile dermoscope attachments that were not compatible with GPs’ new phones. Problems with their imaging equipment can limit GPs from using this equipment or continuing digital dermatology care [11]. Furthermore, a study conducted 10 years ago with the same Dutch teledermoscopy platform already reported equipment issues regarding failing or empty camera batteries and attaching and detaching the dermoscope [41]. Although technology and imaging equipment have developed enormously in recent years, GPs reported that the lack of appropriate up-to-date imaging equipment and equipment costs still hindered their digital dermatology use. Although most GPs own a self-purchased appropriate smartphone device and off-the-shelf dermoscopy attachments are available for a few hundred euros or US dollars, GPs in our study reported that they would appreciate it and consider it as an incentive if imaging equipment would be offered for free by the telemedicine organization. Costs to purchase the imaging equipment was also mentioned in other teledermatology and teledermoscopy studies as a barrier [43-45]. These findings show that the internal organizational policies regarding equipment influence the availability of appropriate hardware and software by GPs and that the lack of use of appropriate equipment directly influences the ability to clinically justify teledermatologists’ advice based on the images. Therefore, solutions for purchasing or hiring up-to-date adequate imaging equipment for GP practices should be considered by telemedicine organizations.

Besides the issues with photography skills and the equipment, the human-computer interface and interoperability issues with the telemedicine platform might have influenced GPs’ intentions of using the digital dermatology negatively. These platform-related issues should be taken into account by the telemedicine organization and might require technological improvements because GPs also reported about missing functionalities in our study. The human-computer interface should be specifically optimized based on the clinical information needs that teledermatologists and GPs have in the digital dermatology decision-making context. For example, the Ksyos digital dermatology platform does not verify whether the photograph’s quality is sufficient or whether the correct number of images are attached. As suggested by GPs in the open-ended questions, quality validation in the consultation platform is needed that allows patients and GPs to only send digital dermatology consultations when all photographs have been taken and uploaded and are with sufficient quality. Such a quality validation step on the platform could warn the user if the uploaded photographs are incomplete or of inadequate quality and could request the GP directly to retake the images. In addition, image quality checklists or guidelines for taking (dermoscopic) images implemented on the platform can instruct GPs and patients to take photographs with sufficient quality [46,47]. Furthermore, a study in the United States showed promising results with an automated machine learning algorithm that evaluates dermatology image quality and provides, if necessary, specific recommendations and guidance to patients about how to improve the quality of their images [48]. Su et al [49] launched a feedback algorithm with “smart phrases” that induces patients to retake images if the latest images were of insufficient quality. Such algorithms might also improve the quality of the submitted images in the Dutch digital dermatology platform over time.

In our study, two-thirds (42/66, 64%) of the GPs agreed that the dermatology care provided through the digital dermatology platform was the same as that in an in-person dermatology visit, meaning that digital consultation could replace regular in-person consultation. This percentage is consistent with a telemedicine study in the United States, where 63% of the physicians responded that the web-based telemedicine quality of care during the pandemic was generally similar to that of in-person care [50]. However, the sociotechnical analysis in our study showed that GPs had different perspectives and reported divergent reasons for when and for which skin conditions and patients they can or cannot apply the digital dermatology service instead of an in-person visit. Furthermore, this variety in GPs’ answers about when they (think they can) apply the service suggest that it is not always clear to GPs which skin conditions are (not) suitable for a digital dermatology consultation. Training by the telemedicine organization and during GP education programs is needed to better instruct GPs when to use and not use the service.

Most GPs in our study responded that they would use the telemedicine platform again (61/66, 92%) and would recommend the platform to a colleague (53/66, 80%). Studies in other countries also show high GP satisfaction with and acceptance levels for digital dermatology care [35,45]. GPs in our study responded that they learned from practical experience (after repeated use of the platform) and the teledermatologist provided feedback, which facilitated the use of the service. This learning curve can be seen as a personal motivator for GPs to apply digital dermatology consultation [11]. Furthermore, this learning curve, in combination with the telemedicine experience level of the GPs before the pandemic, could also have stabilized the number of digital dermatology consultations during the pandemic. In addition, GPs in our study were satisfied with the time-efficient and adequate responses of the dermatologists. A scoping review by Osman et al [51] confirmed that primary care providers’ perspectives about facilitators of digital consultations include obtaining timely responses from specialists and establishing knowledge.

Finally, Dutch GPs generally use digital dermatology consultations to prevent physical referrals, if they are unable to determine a differential diagnosis, if the treatment was unsuccessful, or to receive additional advice from the dermatologist. French GPs also use digital dermatology consultation mostly to resolve diagnostic doubts [52]. Furthermore, approximately two-thirds of these French GPs used the service before the pandemic owing to long waiting times for face-to-face dermatology visits, and one-third of the GPs used the service for emergencies [52]. In our study, approximately one-third (21/66, 32%) of the Dutch GPs mentioned long dermatology waiting times as reason for use, and only 2% (1/66) of the GPs used digital dermatology for emergencies. Thus, both Dutch and French GPs indicate that the use of digital dermatology accelerates contact with dermatologists. Table 5 shows the sociotechnical considerations for remote digital dermatology.

**Table 5:** Sociotechnical considerations for remote digital dermatology.

Barriers	Recommendations for future
GPs’^a^ and patients’ limited digital photography skills	Accredited, continuous, web-based, and good practice training sessions and video instructions as refreshers for GPs (eg, recap about the use of the imaging equipment and the platform) (Video) instructions for GPs and patients about how to obtain adequate photographs Understandable, straightforward, step-by-step guide for GPs and patients
Costs and the lack of appropriate imaging equipment	Solutions for the availability of appropriate imaging equipment (eg, purchasing or hiring up-to-date equipment)
Human-computer interface and interoperability issues	Quality validation in the teledermatology platform that verifies whether the photograph’s quality is sufficient and whether the correct number of images are taken and uploaded Implementing image quality checklists and guidelines about taking (dermoscopic) images
Different use procedures	Policy development about the use of the teledermatology service Training of GPs (by the telemedicine organization and during GP education programs) when they can or cannot use the teledermatology service

^a^GP: general practitioner.

### Strengths

The first strength of this study was the unique opportunity to evaluate GPs’ perspectives and their experienced facilitators and barriers related to the digital dermatology consultation service during the COVID-19 pandemic, as the service had already been integrated into Dutch general practice before the pandemic. These insights are essential to maintain and optimize the quality of digital dermatology services to the needs of the GPs and to stimulate continuous use of the service in the future. The second strength of this study was the use of a sociotechnical model for the interpretation of the data, which has also been used in other telemedicine and telehealth evaluations [26,53,54]. This subsequently allowed us to identify the GPs’ experienced facilitators and barriers related to digital dermatology care. The added value of this model was that it provided insight into the interrelations between the sociotechnical aspects obtained using the SAF-TSUQ and the additional open-ended insight questions. This model has shown that changes and barriers in one of these sociotechnical aspects directly influence the other aspects.

### Limitations

The first limitation of this study is that the questionnaire was distributed to GPs affiliated with the telemedicine organization who performed a store-and-forward consultation between October 2019 and September 2021. In doing so, we excluded GPs working with other telemedicine organizations, GPs who chose not to use the service during the pandemic, or GPs who were less comfortable with using the service. However, the main aim of this study was to assess GPs’ perspectives about 3 types of digital dermatology consultation in the Netherlands. Future studies should expand upon the use of digital dermatology care during the COVID-19 pandemic from the perspectives of other involved stakeholders, such as dermatologists and patients.

The second limitation is that the study data were only collected for 1 already existing Dutch store-and-forward digital dermatology service, even though this service has been implemented nationwide. This may limit the generalizability of our findings to other (Western European) countries that have implemented digital dermatology services, which are still in the preliminary stages. In many countries, teledermatology was not reimbursed before the pandemic, which has driven providers away from practicing teledermatology consultations [6]. Although the telemedicine contexts may differ in other countries, our results also provide general facilitators and barriers that apply to the adoption and implementation of digital dermatology consultation in preliminary stages or other contexts. Future research could involve a more extensive study that would allow us to examine what contextual and other factors (eg, age, the number of years of practice, and the type of practice) influence GPs’ perceptions and use of remote dermatology services.

The third limitation is that our study was questionnaire based, with the typical limitations of incomplete responses and low response rates. The questionnaire was administered at the end of 2021, when a COVID-19 mandated national lockdown, social distancing, and stay-at-home mandates were announced. We assume that the low participation rate might be owing to GPs’ limited time and increased workload. We tried to increase the response rate by sending 1 reminder via email but did not want to burden GPs in such a hectic time.

### Conclusions

Remote dermatology care was already integrated into Dutch GP practice before the pandemic, which may have facilitated the positive responses of GPs to the use of the service. However, barriers impeded the full potential of its successful use by GPs during the pandemic and may limit the continuity of the service in GP practices in the future. The training of GPs is needed to effectively use the imaging equipment and to guarantee adequate quality of taken (dermoscopy) images. Furthermore, GPs should be trained when (not) to use the digital dermatology service. In addition, the remote dermatology platform should be improved to guide patients in taking photographs with sufficient quality. The identification of these barriers provides insights to telemedicine organizations, health institutions, and policy makers to guide digital dermatology implementation and sustainability.

## Appendix

Multimedia Appendix 1 Web-based questionnaire sent to general practitioners and the English translation.

## Abbreviations

GP: general practitioner
NIVEL: Nederlands instituut voor onderzoek van de gezondheidszorg (the Netherlands Institute for Health Services Research)
SAF-TSUQ: Store-and-Forward Telemedicine Service User-satisfaction Questionnaire
