# Supplementary material for: General Practitioners’ Perspectives About Remote Dermatology Care During the COVID-19 Pandemic in the Netherlands: Questionnaire-Based Study
Source: JMIR Dermatol. 2023 Jun 13;6:e46682. doi: 10.2196/46682 (PMC10335141; doi:10.2196/46682)
Supplement: Multimedia Appendix 1 [file derma_v6i1e46682_app1.docx]

# Appendix A: Questionnaire

* Question is mandatory

## General questions

Wat is uw leeftijd?*
*Kies één van de volgende mogelijkheden:*

- 18 t/m 24 jaar
- 25 t/m 34 jaar
- 35 t/m 44 jaar
- 45 t/m 54 jaar
- 55 t/m 64 jaar
- 65 jaar of ouder

What is your age?*

*Choose one of the following options:*

- 18-24 years
- 25-34 years
- 35-44 years
- 45-54 years
- 55-64 years
- ≥ 65 years

Wat is uw geslacht?*
*Kies één van de volgende mogelijkheden:*

- Man
- Vrouw
- Overig
- Wil ik niet delen

What is your sex?*

*Choose one of the following options:*

- Male
- Female
- Other
- Prefer not to say

In welke rol/functie bent u betrokken bij [telemedicine organization]?*

*Meerdere antwoorden mogelijk. Selecteer alle mogelijkheden:*

- Cardioloog
- Dermatoloog
- Doktersassistent
- Huisarts
- Longarts
- Oogarts
- Optometrist
- POH(-GGZ/somatiek)
- Psychiater
- (GZ-)psycholoog of psychotherapeut
- Somnoloog
- Specialist ouderengeneeskunde
- Verpleeghuisarts
- Verslavingsarts
- Verpleegkundig specialist
- Zorgmanager
- Anders, namelijk: …..

In which role/function are you involved in at [telemedicine organization]?*

*Multiple answers possible. Select all that apply:*

- Cardiologist
- Dermatologist
- Physician’s assistant
- General Practitioner
- Pulmonologist
- Ophthalmologist
- Optometrist
- Nurse Practitioner (mental health/somatic)
- Psychiatrist
- (GZ-)Psychologist or psychotherapist
- Somnologist
- Specialist geriatric medicine
- Nursing home physician
- Addiction specialist
- Nurse specialist
- Care manager
- Other, …..

Hoe schat u uw computervaardigheden in?*
*Kies één van de volgende mogelijkheden:*

- Slecht
- Voldoende
- Goed
- Uitstekend

How do you rate your computer skills?*

*Choose one of the following options:*

- Poor
- Sufficient
- Good
- Excellent

Hoe zou u uzelf het beste omschrijven?*
*Kies één van de volgende mogelijkheden:*

- Ik ben een innovator en de eerste die nieuwe technologie wil uitproberen
- Ik ben een pionier en één van de eersten die nieuwe technologie uitprobeert
- Ik ben een voorloper, als anderen nieuwe technologie gebruiken, wil ik het ook gaan gebruiken
- Ik ben een achterloper en meestal één van de laatsten die nieuwe technologie uitprobeert
- Ik ben een achterblijver en de laatste die nieuwe technologie uitprobeert

How would you describe yourself?*

*Choose one of the following options:*

- I am an innovator and the first to try out new technology
- I am an early adopter and am one of the first to try out new technology
- I am a part of the early majority. If others use a new technology, I want to start using it as well.
- I am a part of the late majority and am usually one of the last to try out new technology
- I am a laggard and am the last to try out new technology

Maakt u gebruik van het [telemedicine platform] in de praktijk?*

*Meerdere antwoorden mogelijk. Selecteer alle mogelijkheden:*

- Ja, ik verstuur aanvragen en/of consulten en/of onderzoeken naar een specialist
- Ja, ik beoordeel consulten en/of onderzoeken
- Ja, ik ontvang alleen medische uitslagen van [telemedicine organization] in het Huisarts Informatie Systeem (HIS)
- Ja, ik log in om uitslagen te bekijken
- Ja, ik verstuur online behandelmodules en/of vragenlijsten naar de patiënt
- Nee
- *Weet ik niet*
- Ja, anders

Do you use the [telemedicine platform] in practice?*

*Multiple answers possible. Select all that apply:*

- Yes, I send requests and/or consultations and/or examinations to a specialist
- Yes, I assess consultations and/or examinations
- Yes, I only receive medical results from [telemedicine organization] in the GP information system
- Yes, I log in to view medical results
- Yes, I send web-based treatment modules and/or questionnaires to the patient
- Nee
- *I do not know*
- Yes, other…

Welke dienst(en) gebruikt u van [telemedicine organization]?*
*Meerdere antwoorden mogelijk. Selecteer alle mogelijkheden:*

- Cardiologie
- Dermatologie
- GGZ
- Laboratorium aanvragen
- Oogheelkunde
- Pulmonologie
- Slaap
- Geen van bovenstaande
- *Weet ik niet*

Which service(s) do you use from [telemedicine organization]?*

*Multiple answers possible. Select all that apply:*

- Cardiology
- Dermatology
- Mental health
- Laboratory requests
- Ophthalmology
- Pulmonology
- Sleep
- None of the above
- *I do not know*

Hoe vaak gebruikt u het [telemedicine platform] in de praktijk?*
*Kies één van de volgende mogelijkheden:*

- Dagelijks
- Wekelijks
- Maandelijks
- Een aantal keer per jaar
- Nooit

How often do you use the [telemedicine platform] in practice?*

*Choose one of the following options:*

- Daily
- Weekly
- Monthly
- A few times in a year
- Never

Hoe lang werkt u al met het [telemedicine platform]?*
*Kies één van de volgende mogelijkheden:*

- Minder dan een half jaar
- 6-12 maanden
- 1-3 jaar
- 3-5 jaar
- 5-10 jaar
- Meer dan 10 jaar

How long have you been working with the [telemedicine platform]?*

*Choose one of the following options:*

- <6 months
- 6-12 months
- 1-3 years
- 3-5 years
- 5-10 years
- >10 years

Overige opmerkingen

Other comments

## COVID-19 pandemic

De volgende vragen hebben betrekking op de digitale zorg gedurende de corona pandemie. De eerste corona golf definiëren we als de start van corona (periode maart – mei 2020).

The following questions are related to digital care during the corona pandemic. We define the first corona pandemic as the start of corona (period March – May 2020).

Hoe vaak heeft u het [telemedicine platform] gebruikt in de eerste corona golf vergeleken met de periode voor de corona pandemie?*

*Kies één van de volgende mogelijkheden:*

- Minder vaak gebruikt
- Ongeveer even vaak gebruikt
- Vaker gebruikt
- *Niet van toepassing*

How often have you used the [telemedicine platform] during the first corona wave compared with the period before the corona pandemic?*

*Choose one of the following options:*

- Less often
- Approximately as often
- More often
- *Not applicable*

Hoe vaak gebruikt u het [telemedicine platform] nu?*
*Kies één van de volgende mogelijkheden:*

- Minder vaak dan voor de corona pandemie
- Ongeveer even vaak als voor de corona pandemie
- Vaker dan voor de corona pandemie
- *Niet van toepassing*

How often do you use the [telemedicine platform] currently?*

*Choose one of the following options:*

- Less often than before the corona pandemic
- Approximately as often as before the corona pandemic
  More often than before the corona pandemic
- *Not applicable*

Ontving u voldoende ondersteuning voor het uitvoeren van digitale zorg tijdens de corona pandemie?*
*Kies één van de volgende mogelijkheden:*

- Ja
- Nee

Did you receive sufficient support for carrying out digital care during the corona pandemic?*

*Choose one of the following options:*

- Yes
- No

*Indien “Nee”:*

Wat zou volgens u aan de ondersteuning verbeterd kunnen worden bij een volgende golf en/of pandemie?*

*If “No”:*

What do you think could be improved about support in the event of another wave and/or pandemic?*

Wat heeft u geleerd tijdens de corona pandemie over de toepassing van digitale zorg?*

What have you learned during the corona pandemic about the application of digital care?*

Wat zijn uw ervaringen met het gebruik van het digitale dermatologie consult (regulier, dermatoscopie, thuisconsult) tijdens de corona pandemie?*

*Kies één van de volgende mogelijkheden:*

- Heel negatief
- Negatief
- Neutraal
- Positief
- Heel positief
- Niet van toepassing

What are your experiences with the use of digital dermatology consultation (teledermatology, teledermoscopy, dermatology home consultation) during the corona pandemic?*

*Choose one of the following options:*

- Strongly negative
- Negative
- Neutral
- Positive
- Strongly positive
- Not applicable

*Licht uw ervaring toe:*

*Describe your experience:*

Wat heeft u geleerd van het gebruik van het digitale dermatologie consult (regulier, dermatoscopie, thuisconsult) tijdens de corona pandemie?*

What lessons have you learned from the use of digital dermatology consultation (teledermatology, teledermoscopy, dermatology home consultation) during the corona pandemic?*

Overige opmerkingen

Other comments

## Dermatology home consultation

Heeft u gebruik gemaakt van het [telemedicine organization] dermatologie thuisconsult?*
*Kies één van de volgende mogelijkheden:*

- Ja
- Nee

Have you used the [telemedicine organization] dermatology home consultations?*

*Choose one of the following options:*

- Yes
- No

*Indien thuisconsult “Ja”:*

Wat zijn uw ervaringen met het thuisconsult?*
*Kies één van de volgende mogelijkheden:*

- Heel negatief
- Negatief
- Neutraal
- Positief
- Heel positief

*If dermatology home consultation “Yes”:*

What are your experiences with home consultation?*

*Choose one of the following options:*

- Strongly negative
- Negative
- Neutral
- Positive
- Strongly positive

*Licht uw ervaring toe:*

*Describe your experience:*

*Indien thuisconsult “Ja”:*

Wat vindt u van de kwaliteit van de foto’s die gemaakt zijn door patiënten?*

*Kies één van de volgende mogelijkheden:*

- Altijd slecht
- Meestal slecht
- Af en toe goed, af en toe slecht
- Meestal goed
- Altijd goed

*If dermatology home consultation “Yes”:*

What is your opinion of the quality of the photographs taken by patients?*

*Choose one of the following options:*

- Always poor
- Usually poor
- Sometimes good, sometimes poor
- Usually good
- Always good

*Licht toe waarom u de kwaliteit van de foto’s slecht/goed vindt:*

*Explain why you think that the quality of the photographs is poor/good:*

*Indien thuisconsult “Ja”:*

Voor welke huidaandoeningen is een dermatologie thuisconsult vooral geschikt in uw praktijk?*
*Meerdere antwoorden mogelijk. Selecteer alle mogelijkheden:*

- Rode verkleuring
- Moedervlek
- Bultjes
- Wonden
- Luier uitslag
- Anders, namelijk: …..

If dermatology home consultation “Yes”:

Which skin conditions are especially suitable for a dermatology home consultation in your practice?*

*Multiple answers possible. Select all that apply:*

- Red discoloration
- Birthmark
- Bumps
- Wounds
- Diaper rash
- Other, ….

*Indien thuisconsult “Ja”:*

Voor welke patiënten is het dermatologie thuisconsult het meest geschikt?*

*Meerdere antwoorden mogelijk. Selecteer alle mogelijkheden:*

- Baby's
- Peuters
- Kinderen < 12 jaar
- Kinderen 12 – 18 jaar
- Volwassenen tot 65 jaar
- Volwassenen > 65 jaar
- Anders, namelijk: …..

If dermatology home consultation “Yes”:

For which patients is the dermatology home consultation most suitable?*

*Multiple answers possible. Select all that apply:*

- Babies
- Toddlers
- Children < 12 years
- Children 12 – 18 years
- Adults up to 65 years
- Adults > 65 years
- Other, …..

*Indien thuisconsult “Ja”:*

Heeft u verbeterpunten voor het thuisconsult?* Bijvoorbeeld met betrekking tot foto instructie, technische mogelijkheden, patiënt informatie die bij het thuisconsult aangeleverd wordt, inrichting proces, etc.

If dermatology home consultation “Yes”:

Do you have suggestions for the improvement of home consultations?* For example, regarding photographs instruction, technical options, patient information supplied during home consultation, design of the process, etc.

Kan de patiënt ook op een andere manier digitaal contact met de praktijk opnemen voor zijn huidaandoeningen?*
*Meerdere antwoorden mogelijk. Selecteer alle mogelijkheden:*

- Nee
- Ja, via een (beveiligde) email
- Ja, via beeldbellen
- Ja, via een andere aanbieder
- Anders, namelijk: …..

Could the patient contact the practice digitally by other means for their skin conditions?*

*Multiple answers possible. Select all that apply:*

- No
- Yes, via a (secured) email
- Yes, via video calling
- Yes, via another supplier
- Other, …..

Overige opmerkingen

Other comments

## Training

|  |  | Helemaal oneens | Oneens | Neutraal | Eens | Helemaal eens | *Weet ik niet* | *Niet van toepassing* |
| --- | --- | --- | --- | --- | --- | --- | --- | --- |
| Q1 | De training en uitleg die door [telemedicine organization] wordt aangeboden zijn voldoende om het [telemedicine platform] te kunnen gebruiken in mijn werkzaamheden* | ⃝ | ⃝ | ⃝ | ⃝ | ⃝ | ⃝ | ⃝ |
| Q2 | Ik ben tevreden met de mogelijkheden voor bij- en/of nascholing die door [telemedicine organization] worden aangeboden* | ⃝ | ⃝ | ⃝ | ⃝ | ⃝ | ⃝ | ⃝ |
| Q3 | Ik ben tevreden met de taken die ik uitvoer binnen mijn functie in het [telemedicine platform]* | ⃝ | ⃝ | ⃝ | ⃝ | ⃝ | ⃝ | ⃝ |
| Q4 | Door het [telemedicine platform] te gebruiken ben ik in staat om mijn kennis en vaardigheden genoeg te ontwikkelen* | ⃝ | ⃝ | ⃝ | ⃝ | ⃝ | ⃝ | ⃝ |
| Q5 | Ik ben van mening dat het [telemedicine platform] aan mijn wensen en verwachtingen voor digitale zorgverlening voldoet* | ⃝ | ⃝ | ⃝ | ⃝ | ⃝ | ⃝ | ⃝ |

Met taken wordt bedoeld het versturen van aanvragen en/of consulten en/of onderzoeken naar een specialist, het beoordelen van consulten/onderzoeken, het ontvangen of bekijken van medische uitslagen, het versturen van online behandelmodules en/of vragenlijsten naar de patiënt, etc.

Met “kennis en vaardigheden ontwikkelen” wordt bedoeld in hoeverre u digitale en medische kennis verkrijgt door te werken met het telemedicine platform.

|  |  | Strongly disagree | Disagree | Neutral | Agree | Strongly agree | *I do not know* | *Not applicable* |
| --- | --- | --- | --- | --- | --- | --- | --- | --- |
| Q1 | The training and explanation offered by [telemedicine organization] are sufficient to be able to use the [telemedicine platform] in my work* | ⃝ | ⃝ | ⃝ | ⃝ | ⃝ | ⃝ | ⃝ |
| Q2 | I am satisfied with the possibilities for additional and/or continuing education offered by [telemedicine organization]* | ⃝ | ⃝ | ⃝ | ⃝ | ⃝ | ⃝ | ⃝ |
| Q3 | I am satisfied with the tasks I perform within my profession in the [telemedicine platform]* | ⃝ | ⃝ | ⃝ | ⃝ | ⃝ | ⃝ | ⃝ |
| Q4 | By using the [telemedicine platform] I am able to develop my knowledge and skills adequately* | ⃝ | ⃝ | ⃝ | ⃝ | ⃝ | ⃝ | ⃝ |
| Q5 | I believe that [telemedicine platform] meets my wishes and expectations for telemedicine* | ⃝ | ⃝ | ⃝ | ⃝ | ⃝ | ⃝ | ⃝ |

*‘Tasks’ refers to sending requests and/or consultations and/or examinations to a specialist, assessing consultations and/or examinations, receiving or viewing medical results, sending web-based treatment modules and/or questionnaires to the patient, etc.*

*‘Developing knowledge and skills’ refers to the extent to which you acquire digital medical knowledge by working with the [telemedicine platform].*

Overige opmerkingen

Other comments

## Communication

|  |  | Helemaal oneens | Oneens | Neutraal | Eens | Helemaal eens | *Weet ik niet* | *Niet van toepassing* |
| --- | --- | --- | --- | --- | --- | --- | --- | --- |
| Q6 | De informatie en handleidingen die vanuit [telemedicine organization] aan mij worden verstrekt en de instructies die ik heb ontvangen om mijn werk uit te voeren zijn voldoende* | ⃝ | ⃝ | ⃝ | ⃝ | ⃝ | ⃝ | ⃝ |
| Q7 | Ik ontvang voldoende relevante informatie met betrekking tot de besluiten, projecten en activiteiten van [telemedicine organization] die invloed op mij kunnen hebben* | ⃝ | ⃝ | ⃝ | ⃝ | ⃝ | ⃝ | ⃝ |
| Q8 | Ik weet op welke manier ik contact kan opnemen met [telemedicine organization] indien ik vragen heb over mijn werk voor [telemedicine organization]* | ⃝ | ⃝ | ⃝ | ⃝ | ⃝ | ⃝ | ⃝ |
| Q9 | Er zijn genoeg informatie kanalen voor suggesties of klachten bij [telemedicine organization]* | ⃝ | ⃝ | ⃝ | ⃝ | ⃝ | ⃝ | ⃝ |

|  |  | Strongly disagree | Disagree | Neutral | Agree | Strongly agree | *I do not know* | *Not applicable* |
| --- | --- | --- | --- | --- | --- | --- | --- | --- |
| Q6 | The information and manuals that are provided to me by [telemedicine organization] and the instructions given to perform my work are sufficient* | ⃝ | ⃝ | ⃝ | ⃝ | ⃝ | ⃝ | ⃝ |
| Q7 | I receive sufficient relevant information regarding any decisions, projects and activities of [telemedicine organization] that may affect me* | ⃝ | ⃝ | ⃝ | ⃝ | ⃝ | ⃝ | ⃝ |
| Q8 | I know how to contact [telemedicine organization] if I have questions about my work for [telemedicine organization]* | ⃝ | ⃝ | ⃝ | ⃝ | ⃝ | ⃝ | ⃝ |
| Q9 | There are sufficient information channels for suggestions or complaints at [telemedicine organization]* | ⃝ | ⃝ | ⃝ | ⃝ | ⃝ | ⃝ | ⃝ |

Overige opmerkingen

Other comments

## Interaction telemedicine platform

|  |  | Helemaal oneens | Oneens | Neutraal | Eens | Helemaal eens | *Weet ik niet* | *Niet van toepassing* |
| --- | --- | --- | --- | --- | --- | --- | --- | --- |
| Q23 | Ik heb genoeg en de juiste middelen (zoals computers, holterkastjes, gebruiksvoorwerpen, etc.) om mijn (dagelijkse) werk voor [telemedicine organization] uit te kunnen voeren* | ⃝ | ⃝ | ⃝ | ⃝ | ⃝ | ⃝ | ⃝ |
| Q24 | Het is gemakkelijk om het [telemedicine platform] te gebruiken* | ⃝ | ⃝ | ⃝ | ⃝ | ⃝ | ⃝ | ⃝ |
| Q27 | Ik vind het prettig om het [telemedicine platform] te gebruiken* | ⃝ | ⃝ | ⃝ | ⃝ | ⃝ | ⃝ | ⃝ |
| Q28 | Het [telemedicine platform] is simpel en gemakkelijk te begrijpen* | ⃝ | ⃝ | ⃝ | ⃝ | ⃝ | ⃝ | ⃝ |
| Q29 | Het [telemedicine platform] bevat alle functionaliteiten die ik zou verwachten* | ⃝ | ⃝ | ⃝ | ⃝ | ⃝ | ⃝ | ⃝ |
| Q30 | Wanneer ik binnen het [telemedicine platform] een fout maak kan ik deze gemakkelijk en snel herstellen* | ⃝ | ⃝ | ⃝ | ⃝ | ⃝ | ⃝ | ⃝ |
| Q31 | Het [telemedicine platform] geeft foutmeldingen die duidelijk vermelden hoe ik problemen kan oplossen* | ⃝ | ⃝ | ⃝ | ⃝ | ⃝ | ⃝ | ⃝ |

|  |  | Strongly disagree | Disagree | Neutral | Agree | Strongly agree | *I do not know* | *Not applicable* |
| --- | --- | --- | --- | --- | --- | --- | --- | --- |
| Q23 | I have sufficient and appropriate resources (such as computers, holterdevices, equipment, etc.) to perform my (daily) work for [telemedicine organization]* | ⃝ | ⃝ | ⃝ | ⃝ | ⃝ | ⃝ | ⃝ |
| Q24 | It is easy to use the [telemedicine platform]* | ⃝ | ⃝ | ⃝ | ⃝ | ⃝ | ⃝ | ⃝ |
| Q27 | I like using the [telemedicine platform]* | ⃝ | ⃝ | ⃝ | ⃝ | ⃝ | ⃝ | ⃝ |
| Q28 | The [telemedicine platform] is simple and easy to understand* | ⃝ | ⃝ | ⃝ | ⃝ | ⃝ | ⃝ | ⃝ |
| Q29 | The [telemedicine platform] includes all functionalities I would expect* | ⃝ | ⃝ | ⃝ | ⃝ | ⃝ | ⃝ | ⃝ |
| Q30 | Whenever I make a mistake within the [telemedicine platform] I can easily and quickly fix it* | ⃝ | ⃝ | ⃝ | ⃝ | ⃝ | ⃝ | ⃝ |
| Q31 | The [telemedicine platform] provides error messages that clearly state how I can fix problems* | ⃝ | ⃝ | ⃝ | ⃝ | ⃝ | ⃝ | ⃝ |

Overige opmerkingen

Other comments

## Use of telemedicine platform

|  |  | Helemaal oneens | Oneens | Neutraal | Eens | Helemaal eens | *Weet ik niet* | *Niet van toepassing* |
| --- | --- | --- | --- | --- | --- | --- | --- | --- |
| Q32 | Het [telemedicine platform] verbetert de toegang tot de gezondheidszorg* | ⃝ | ⃝ | ⃝ | ⃝ | ⃝ | ⃝ | ⃝ |
| Q33 | Het [telemedicine platform] biedt mij wat ik nodig heb om betere zorg te leveren | ⃝ | ⃝ | ⃝ | ⃝ | ⃝ | ⃝ | ⃝ |
| Q34 | Ik werd snel productief door het werken met het [telemedicine platform]* | ⃝ | ⃝ | ⃝ | ⃝ | ⃝ | ⃝ | ⃝ |
| Q35 | Ik ben van mening dat de dermatologie zorg die via het [telemedicine platform] wordt geleverd hetzelfde is als een regulier consult^+^* | ⃝ | ⃝ | ⃝ | ⃝ | ⃝ | ⃝ | ⃝ |
| Q36 | Ik ben van plan het [telemedicine platform] nogmaals te gebruiken* | ⃝ | ⃝ | ⃝ | ⃝ | ⃝ | ⃝ | ⃝ |
| Q37 | Ik zou het [telemedicine platform] aanraden aan een collega* | ⃝ | ⃝ | ⃝ | ⃝ | ⃝ | ⃝ | ⃝ |

^+^Hiermee bedoelen we vindt u dat een digitaal consult een regulier consult kan vervangen?

|  |  | Strongly disagree | Disagree | Neutral | Agree | Strongly agree | *I do not know* | *Not applicable* |
| --- | --- | --- | --- | --- | --- | --- | --- | --- |
| Q32 | The [telemedicine platform] improves access to healthcare services* | ⃝ | ⃝ | ⃝ | ⃝ | ⃝ | ⃝ | ⃝ |
| Q33 | The [telemedicine platform] offers me what I need to deliver better care* | ⃝ | ⃝ | ⃝ | ⃝ | ⃝ | ⃝ | ⃝ |
| Q34 | I quickly became productive while working with the [telemedicine platform]* | ⃝ | ⃝ | ⃝ | ⃝ | ⃝ | ⃝ | ⃝ |
| Q35 | I believe that the dermatology care provided through the [telemedicine platform] is the same as an in-person consultation^+^* | ⃝ | ⃝ | ⃝ | ⃝ | ⃝ | ⃝ | ⃝ |
| Q36 | I would use the [telemedicine platform] again* | ⃝ | ⃝ | ⃝ | ⃝ | ⃝ | ⃝ | ⃝ |
| Q37 | I would recommend the [telemedicine platform] to a colleague* | ⃝ | ⃝ | ⃝ | ⃝ | ⃝ | ⃝ | ⃝ |

^+^We mean do you think that a digital consultation can replace a regular consultation?

Overige opmerkingen

Other comments

## Use of digital dermatology consultation (teledermatology and teledermoscopy) in general practice

Wanneer zet u digitale dermatologie consultatie in in uw dagelijkse praktijk?*
*Meerdere antwoorden mogelijk. Selecteer alle mogelijkheden:*

- Voorkómen fysieke verwijzing
- Extra advies
- Spoedgevallen
- Lange wachttijden in het ziekenhuis
- Op verzoek van de patiënt
- Lagere kosten voor de patiënt
- Bij verdenking op maligniteit
- Als ik er bij de differentiaal diagnose niet uit kom
- Als de behandeling niet aanslaat
- Als ik twijfel over de grootte van de afwijking
- Anders, namelijk: …..

When do you use digital dermatology consultation in your daily practice?*

*Multiple answers possible. Select all that apply:*

- Preventing physical referrals
- Receiving additional advice
- Emergencies
- Long waiting times in hospitals
- At the request of the patient
- Lower costs for the patient
- Suspicion of malignancy
- Unable to determine a differential diagnosis
- Treatment is unsuccessful
- Doubts about the size of the deviation
- Other, …..

Wanneer zet u digitale dermatologie consultatie niet in in uw dagelijkse praktijk?*

When do you not use digital dermatology consultation in your daily practice?*

Maakt u bij een digitaal dermatologie consult zelf (dermatoscopische) foto’s van de huid of laat u dit meestal doen door een POH of assistent?*
*Kies één van de volgende mogelijkheden:*

- Ja, ik maak de dermatologische foto’s zelf
- Nee, ik laat de foto’s maken door een POH of assistent
- Anders, namelijk: …..

During a digital dermatology consultation, do you take (dermoscopic) photographs of the skin yourself or does a nurse practitioner or assistant do this for you?*

*Choose one of the following options:*

- Yes, I take the dermatological photographs myself
- No, a nurse practitioner or assistant takes the photographs
- Other, …..

Heeft u wel eens feedback gehad van de dermatoloog over de kwaliteit van uw foto’s?*
*Kies één van de volgende mogelijkheden:*

- Ja
- Nee

Have you ever received feedback from the dermatologist about the quality of your photographs?*

*Choose one of the following options:*

- Yes
- No

*Indien feedback “Ja”:*

Waar ging de feedback over?*

*If feedback “Yes”:*

What was the feedback about?*

Zijn er volgens u nog verbeterpunten voor het optimaliseren van de foto kwaliteit?*

Do you think there are still areas of improvement for optimizing the photograph quality?*

Hoe zeker bent u in het vaststellen van het behandel beleid van de patiënt na afloop van een regulier digitaal dermatologie of dermatoscopie consult (obv het ontvangen advies en de diagnose van de dermatoloog)?*
*Kies één van de volgende mogelijkheden:*

- Helemaal niet zeker
- Niet zeker
- Neutraal
- Zeker
- Zeer zeker

How confident are you in determining the patient’s treatment policy after a teledermatology or teledermoscopy consultation (based on the advice and the diagnosis received from the dermatologist)?*

*Choose one of the following options:*

- Not sure at all
- Unsure
- Neutral
- Sure
- Very sure

Heeft u een training of uitleg gehad over het maken van (dermatoscopische) foto’s?*

*Meerdere antwoorden mogelijk. Selecteer alle mogelijkheden:*

- Ja, ik heb een uitleg via [telemedicine organization] gehad
- Ja, ik heb nog een (aanvullende) cursus gevolgd
- Ja, ik heb dit geleerd tijdens mijn opleiding tot huisarts
- Ja, ik heb ervaring opgedaan in de praktijk
- Nee, ik heb geen uitleg gehad en ik vind het ook niet nodig
- Nee, ik heb geen uitleg gehad en ik zou het wel gehad willen hebben

Have you received any training or instruction about taking (dermoscopic) photographs?*

*Multiple answers possible. Select all that apply:*

- Yes, I received an instruction via the [telemedicine organization]
- Yes, I attended a(n) (additional) course
- Yes, I learned this during my training as a general practitioner
- Yes, I gained experience in daily practice
- No, I did not receive any instruction and I do not think it is necessary
- No, I did not receive any instruction and I wish I had received it

*Indien (aanvullende) cursus gevolgd:*

Welke (aanvullende) cursus heeft u gevolgd?*

*If attended (additional) course:*

Which (additional) course have you attended?*

Overige opmerkingen

Other comments

## Questionnaire improvement

We willen deze vragenlijst en onze dienstverlening blijven verbeteren. We horen dan ook graag wat u van de vragenlijst vindt. Mist u nog iets of heeft u nog opmerkingen/tips?

We want to keep improving this questionnaire and our services. Therefore we would like to hear what you think of the questionnaire. Is there something missing or do you have any comments/tips?
